# PeptiCKDdb—peptide- and protein-centric database for the investigation of genesis and progression of chronic kidney disease

**DOI:** 10.1093/database/baw128

**Published:** 2016-09-01

**Authors:** Magdalena Krochmal, Marco Fernandes, Szymon Filip, Claudia Pontillo, Holger Husi, Jerome Zoidakis, Harald Mischak, Antonia Vlahou, Joachim Jankowski

**Affiliations:** 1 Biomedical Research Foundation Academy of Athens, Center of Basic Research, Athens, Greece; 2 University Hospital RWTH Aachen University, Institute for Molecular Cardiovascular Research, Aachen, Germany; 3 University of Glasgow, BHF Glasgow Cardiovascular Research Centre, Glasgow, United Kingdom; 4 Experimental Nephrology and Hypertension, Charité—Universitätsmedizin Berlin, Berlin, Germany; 5 Mosaiques Diagnostics GmbH, Hannover, Germany; 6 University of Glasgow, Institute of Cardiovascular and Medical Sciences, Glasgow, United Kingdom; 7 University of Maastricht, CARIM School for Cardiovascular Diseases, Maastricht, The Netherlands

## Abstract

The peptiCKDdb is a publicly available database platform dedicated to support research in the field of chronic kidney disease (CKD) through identification of novel biomarkers and molecular features of this complex pathology. PeptiCKDdb collects peptidomics and proteomics datasets manually extracted from published studies related to CKD. Datasets from peptidomics or proteomics, human case/control studies on CKD and kidney or urine profiling were included. Data from 114 publications (studies of body fluids and kidney tissue: 26 peptidomics and 76 proteomics manuscripts on human CKD, and 12 focusing on healthy proteome profiling) are currently deposited and the content is quarterly updated. Extracted datasets include information about the experimental setup, clinical study design, discovery-validation sample sizes and list of differentially expressed proteins (*P*-value < 0.05). A dedicated interactive web interface, equipped with multiparametric search engine, data export and visualization tools, enables easy browsing of the data and comprehensive analysis. In conclusion, this repository might serve as a source of data for integrative analysis or a knowledgebase for scientists seeking confirmation of their findings and as such, is expected to facilitate the modeling of molecular mechanisms underlying CKD and identification of biologically relevant biomarkers.

**Database URL:** www.peptickddb.com

## Introduction

Chronic kidney disease (CKD), a global public health problem, is a condition characterized by a gradual loss of kidney function over time (1). CKD comprises a group of glomerular diseases and is associated with and/or caused by comorbidities such as diabetes mellitus or hypertension, therefore the pathophysiology is very complex. CKD is a highly heterogeneous disease with regards to the cause of pathology, extent of kidney damage, progression rate and co-existing conditions (2). Glomerular filtration rate (GFR [mL/min/1.73 m^2^]) is an established method for assessment of kidney function, whereas albuminuria and proteinuria are indicative of kidney damage (3). Nevertheless, current clinical biomarkers are of low predictive value, especially in the early stages of the disease and thus, only advanced kidney dysfunction is accurately diagnosed, when treatment options are already limited (4). Moreover, given the complexity of disease processes and the multiple factors that affect progression over time, there is a great need for novel biomarkers that might provide insights into disease mechanisms and improve diagnosis and prognosis of patients. The advent of high throughput approaches, such as genomics, transcriptomics, proteomics and metabolomics, has stimulated many efforts to establish biomarkers or biomarker panels of renal disease.

Analysis of human proteome provides a ‘snapshot’ of the current state of the organism, reflecting the influence of diet, drugs or disease. Through comparison of healthy and diseased proteomes, deregulated molecular pathways can be identified, consequently leading to the elucidation of pathological mechanisms and the discovery of disease-associated proteins (5). Mass spectrometry-based (MS) proteomics enables the investigation of the human proteome in a variety of specimens such as tissue or body fluids (e.g. plasma, urine, saliva). Many putative biomarkers of kidney disease have been proposed using these approaches (6). However, high-throughput data resulting from MS experiments are often underexploited due to the high number of potential targets of interest.

Therefore, data mining and integration strategies aim at offering novel insights through collective analysis of information gathered from multiple sources, forming a unified view and ultimately, a better understanding of the studied system, phenomenon etc. An increasing number of scientific publications and high-throughput datasets do not contribute significantly to scientific advances, because they are not supported by data curators, databases and bioinformatics analyses (7). Hence, curation and sharing of the data among the scientific community is crucial, as it assures data usability, facilitates integration, encourages collaboration and thus, leads to novel discoveries (8). Specialized scientific databases of curated information, which have proliferated over the last decade, largely facilitate this process and contribute to knowledge generation (9).

Presently, three omics databases exist in the field of kidney disease, namely, ‘Chronic Kidney Disease database’ (CKDdb) (10), the ‘Human Urinary Proteome Fingerprint Database’ (UPdb) (11) and ‘The Kidney and Urinary Pathway Knowledge Base’ (KUPKB) (12), providing public access to existing peptidomics or proteomics datasets and mining tools (13). However, both multi-omics resources CKDdb and KUPKB, completely lack data provided from peptidomic approaches, whereas UPdb stores mass peak information (*m*/*z* values), but peptide or protein information is rarely assigned. Therefore, currently there is no database focusing specifically on a comprehensive representation of peptide sequences and proteomics data that would enable rapid and interactive evaluation of deposited data. The aim of the current project was the development of an open-source peptide- and protein-centric resource, collecting datasets extracted from the state-of-the-art publications related to chronic kidney disease (CKD). To the best of our knowledge, the peptiCKDdb is a unique resource that puts emphasis on accurate peptidomics and proteomics data representation and utilization, through comprehensive design of the database structure to encompass all the data- and study-specific information and through implementation of an advanced search engine to perform complex data queries. An interactive graphical interface equipped with table manipulation tools (filtering, ordering and data export) and responsive charts to visualize data, allows for efficient and intuitive exploration of the data and ‘at-a-glance’ interpretation of query results. This repository should serve as a knowledge base for scientists seeking confirmation of their findings or a source of data for integrative analysis and as such, is expected to facilitate the modeling of molecular mechanisms underlying CKD and contribute to identification of biologically relevant biomarkers.

## Materials and Methods

### Literature mining

**Figure 1.**
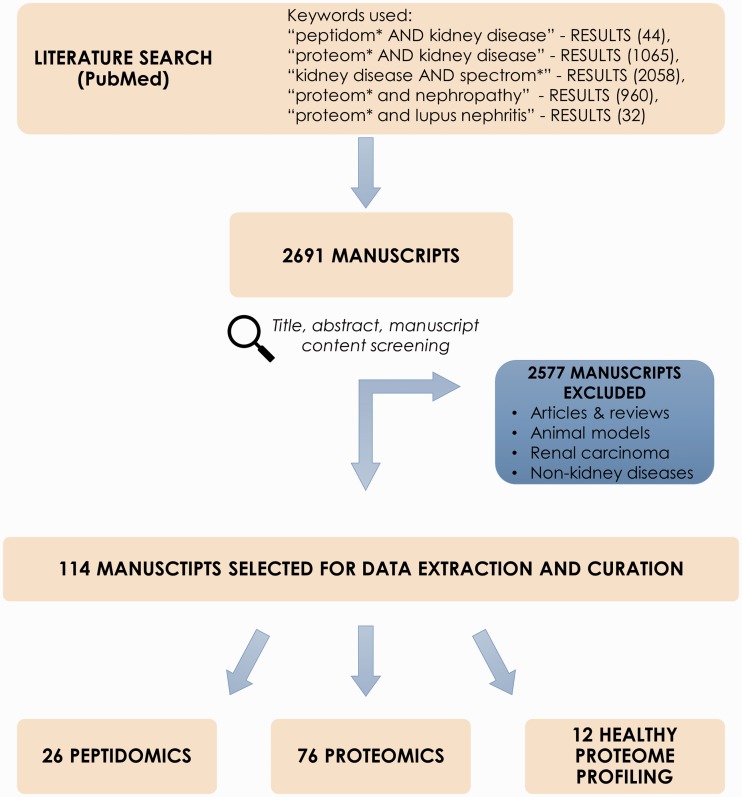
Process of literature mining for retrieval of CKD-relevant manuscripts.

Collection of the peptidomics and proteomic datasets relevant to chronic kidney disease (CKD) was initiated via screening of scientific literature resource, i.e. PubMed (www.ncbi.nlm.nih.gov/pubmed). Literature search was performed using keywords: ‘peptidom* AND kidney disease’, ‘proteom* AND kidney disease’, ‘biomarker AND kidney disease’, ‘kidney disease AND spectrom*’, ‘proteom* and nephropathy’, ‘proteom* and lupus nephritis’ (search performed 10/03/16). Reviews and non-English manuscripts were excluded from further review. Screening and curation was performed by two curators. Query results were checked in accordance to several inclusion criteria, i.e. peptidomics or proteomics case–control study relative to CKD, only human subjects. Of interest were studies focusing on CKD or other related conditions that affect the kidneys, such as diabetes mellitus, hypertension, glomerulonephritis, polycystic kidney disease (PKD), lupus nephritis (LN) or kidney stones. Systematic reviews, articles and manuscripts out of scope of the project (e.g. renal carcinoma, animal models and non-kidney diseases) were excluded. Initial screening of manuscripts was based on the title and abstract. Consecutively, manuscript content was checked for availability of information on differentially expressed peptides/proteins. Importantly, information about peptide sequence/protein identification was required to select the manuscript for data curation. In order to complement the literature search and identify articles that did not appear in PubMed search results, publicly available -omics databases related to kidney pathologies, i.e. the ‘Chronic Kidney Disease database’ (CKDdb; www.padb.org/ckddb) (10), ‘The Kidney and Urinary Pathway Knowledge Base’ (KUPKB; www.kupkb.org) (12) and the ‘Human Urinary Proteome Fingerprint Database’ (UPdb; www.padb.org/updb) (11) were screened. A subset of manuscripts in compliance with inclusion criteria was subjected to data extraction. Figure 1 shows the process of literature mining for retrieval of CKD-relevant manuscripts.

### Database design

**Figure 2.**
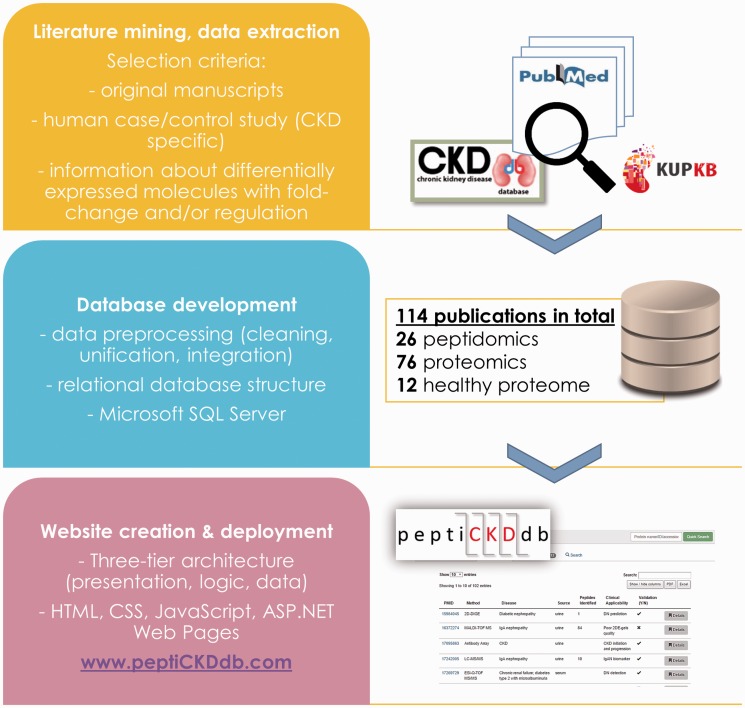
Steps followed in the database development workflow.

The general steps followed in the process of the database development are presented in Figure 2. Database structure was designed based on the relational database model. To ensure precise extraction of study-specific information and representation of proteomics and peptidomics data an Entity-Relationship (ER) model was constructed and tested. Simple representation of database structure is presented on ER diagram (Supplementary Figure S1). The design was based on seven separate entities (tables):

- Publication Information—information about the manuscript (title, authors, journal);
- Experimental Information—experiment setting, peptidomics/proteomics platform used in the experiment, disease etiology, existence of validation;
- Clinical Data—cohort information, number and type of cases/controls, sample types, sample preparation method;
- Validation—information about validation setting, applied method(s), number of samples;
- Proteomics—differentially expressed proteins extracted from selected manuscripts, if available fold change/ratio/regulation and exact p-value was denoted;
- Peptidomics—differentially expressed peptides extracted from selected manuscripts, if available fold change/ratio/regulation and exact p-value was denoted;
- UniProtDB—reference database for protein annotation (UniProt accession number (AC), UniProt ID). Peptides were annotated based on the corresponding parent protein.

Database was implemented in Microsoft SQL Server (MS SQL Server 2014).

### Data extraction, pre-processing and loading

For selected manuscripts, data regarding the study design and statistically significant results (*P*-value < 0.05, as denoted in each manuscript) were manually curated. Each manuscript was thoroughly screened in order to identify all information in accordance with the database structure, i.e. experiment setting, cohort and samples, validation, differentially expressed molecules etc. Data pre-processing was performed to assure common format of identifiers and high consistency of the extracted data. Pre-processing steps involved data cleaning, i.e. mapping of identifiers to one common format, i.e. publicly available UniProt ID, therefore if a different type of protein identifier was used in the manuscript (e.g. IPI), its UniProt equivalent was assigned. Peptides were annotated based on the UniProt ID of the corresponding parent protein. Moreover, if fold change/ratio were available, protein was assigned a regulation trend (UP/DOWN). Finally, all datasets of differentially expressed, statistically significant proteins (*P*-value < 0.05) were deposited into the database.

### Web interface development

Web interface was developed to enable fast querying and retrieval of the stored data and online access to the database. Web application design was based on the three tier architecture consisting of three layers, i.e. application, logic and data. Application tier (presentation interface) was implemented in HTML, CSS and JavaScript with support of Bootstrap framework (www.getbootstrap.com) for responsive design and layouts and DataTables plug-in for tables’ manipulation (www.datatables.net). Interactive charts were created with use of amCharts library (www.amcharts.com). Logic tier (business logic) enabling data retrieval was implemented in ASP.NET Web Pages. Data tier (database) was implemented and managed in MS SQL Server 2014. Main features of the interface include easy data browsing, data export features (to XLS/CSV/PDF data formats), quick and multiparametric search and query results visualizations.

## Results

### Literature mining and curation 

Literature mining in PubMed resulted in 2691 manuscripts. After exclusion of reviews, non-CKD related studies, animal models etc. (2577 entries excluded) abstracts were further checked for adequacy with regards to aforementioned criteria. Consequently, 114 manuscripts were retrieved and selected for data extraction and curation; these included 26 peptidomics and 76 proteomic publications related to CKD and 12 manuscripts describing healthy proteome profiling. Studied sample types include body fluids, i.e. urine, blood, peritoneal dialysate and kidney tissue. Noticeably, urine-based experiments consist of almost 70% of all deposited data.

Extracted data cover 30 distinct kidney diseases or kidney conditions, with majority of manuscripts focusing on diabetic nephropathy (18%), IgA nephropathy (10%), CKD (10%), polycystic kidney disease (7%) (Supplementary Table S1). A total of 415 non-redundant peptide sequences (corresponding to 77 unique proteins) originate from the peptidomics studies. Furthermore, 4225 unique protein identifications extracted from shotgun proteomics experiments are deposited in the database. Among the most represented peptides in the database [based on the total of 26 peptidomics manuscripts] are sequences of collagen alpha-1(I), collagen alpha-1(III), alpha-1-antitrypsin and uromodulin. Most frequent protein findings from proteomics experiments (88 studies) include Ig kappa chain V-IV, serum albumin, kininogen-1 and protein AMBP. Similar results are obtained when comparing frequency distribution of peptides and proteins in different reports (detailed list of the most represented molecules in the database can be found in the Supplementary Tables S3–S7).

### Database features

The peptiCKDdb is developed to facilitate access to proteomics findings in the field of CKD, and therefore support research efforts in this area. The main goal of the designed interface was to enable user-friendly access to data. The major functionalities implemented in the interface are easy records browsing, search tools to perform complex queries on the data and export/visualization features.

#### Records browsing

**Figure 3.**
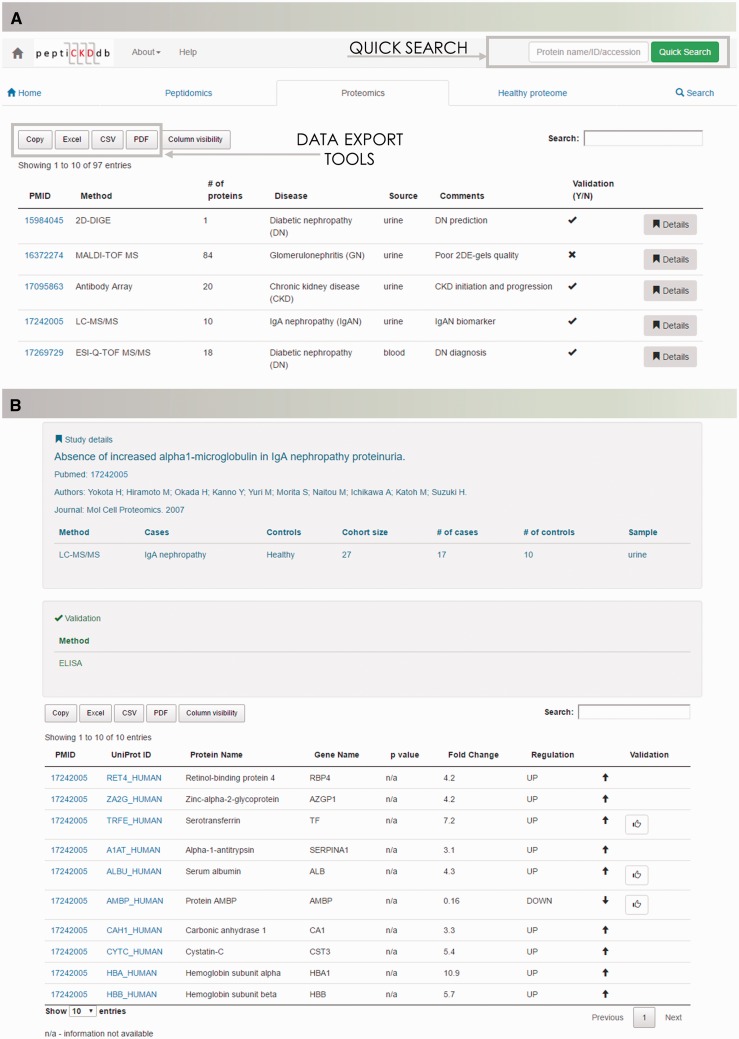
The peptiCKDdb database functionality—record browsing. (A) View of the interface for records browsing and (B) screenshot of the Details view presenting information extracted from one manuscript.

Database content is presented with regards to type of analysis (peptidomics, proteomics and healthy proteome profiling studies). Disease-specific peptidomics and proteomics, and healthy proteome dataset summaries are displayed in tables on separate tabs for optimal transparency (Figure 3A). Each table is equipped with a search option to quickly shortlist studies of interest. Lists can be exported to Microsoft Excel, CSV or PDF formats. Each dataset contains detailed information on the study setup (discovery/validation method used, analyzed sample type) and clinical cohort information (cases, controls and cohort size) and a dataset. In the table, data of differentially expressed molecules, *P*-value and regulation are given (Figure 3B).

#### Data retrieval

**Figure 4.**
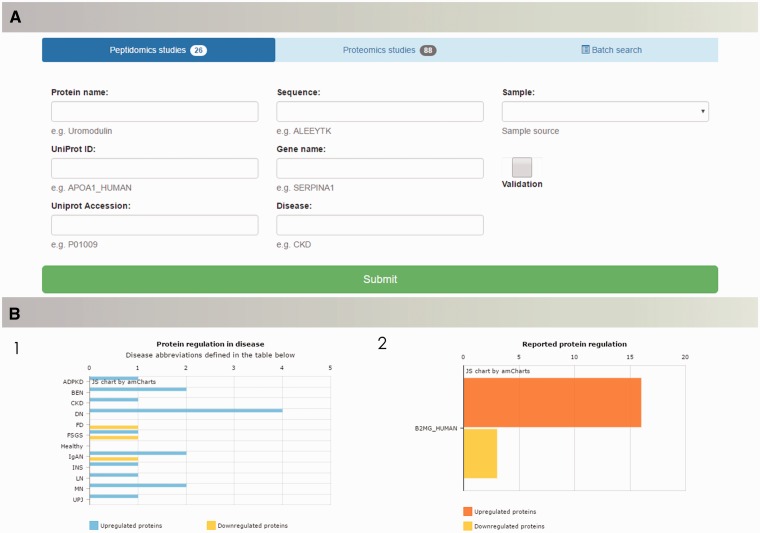
The peptiCKDdb database functionality—multiparametric search allows for selection of specific query criteria, such as protein name/ID/sequence, disease, sample type, present validation (A). Query results are visualized in form of graphs (1) showing distribution of proteins yielded in search among different diseases and (2) collective regulation of proteins in different studies (B).

Search capabilities, implemented to enable retrieval of the data from the database, offer a range of possibilities to query the database. ‘Quick Search’ allows for fast retrieval of information about peptides and proteins of interest by entering protein name, UniProt ID, UniProt AC or gene name. Multi-parametric search was designed to handle complex queries and precisely set the search criteria. Users of the database are able to browse the literature data based on the protein name (UniProt ID/AC), peptide sequence in case of peptidomics data, gene name, specific disease or sample type. Batch search enables search for multiple peptide sequences or proteins of interest (Figure 4A). Query results are displayed in the form of searchable tables, with possibility of manipulation (column/content ordering, columns’ visibility and data export).

#### Visualization

Query results are visualized in the form of charts in order to facilitate interpretation of the results (Figure 4B). For each search, query output is presented as occurrence of searched protein(s) per disease and collectively reported regulation (UP/DOWN) for all results.

#### Export features

Studies lists, extracted datasets as well as query results can be easily exported to Microsoft Excel, CSV or PDF file formats. Subsequently, datasets can be further analyzed or integrated with the use of external tools.

## Data Exploration

Multiple possible applications of database utility in data exploration can be listed. As an example, analysis of the peptide sequences with tools for protease prediction such as Proteasix (14) might be helpful in prediction of molecular mechanisms of disease and prediction of drug targets.

Moreover, through comparison of urine and kidney proteome we can identify proteins of intracellular origin present in urine, which may be of interest when studying disease-specific processes. Using deposited data, we compared 750 differentially expressed proteins identified in urine and 553 unique proteins found in the kidney tissue (proteomics experiments). We present an overlap of 177 proteins (15.7%), for which cellular localization was reported (data from UniProt database). In the overlapping protein fraction ≈63% of molecules were secreted and ≈37% were found to be intracellular proteins (Supplementary Table S2).

Analysis of proteins regulation with regards to sample type revealed consistent regulation trend in 74.8% of urinary proteins, 93.6% of kidney proteins in kidney and 91.9% of blood proteins. Therefore, we can notice that regulation of proteins in blood and kidney is consistent, yet the reproducibility in urine is lower (Supplementary Figure S2). Therefore, when exploring the regulation trends it is important to take into account the variability of the protein expression within different disease etiologies, which can offer further insights into disease-associated activities. Examples of such disease-specific regulation trends for IgA nephropathy (IgAN) and diabetic nephropathy (DN) presented in the Supplementary data (Supplementary Figures S3 and S4, Supplementary Tables S8 and S9).

Given the plethora of studies aiming at the description of a healthy human proteome, we combined the identified molecules in an attempt to define urinary proteome of healthy subjects. In addition, we performed the comparison of proteins identified in urine and exosomes, which showed an overlap of 32.3% between both groups (Supplementary Table S10).

**Figure 5.**
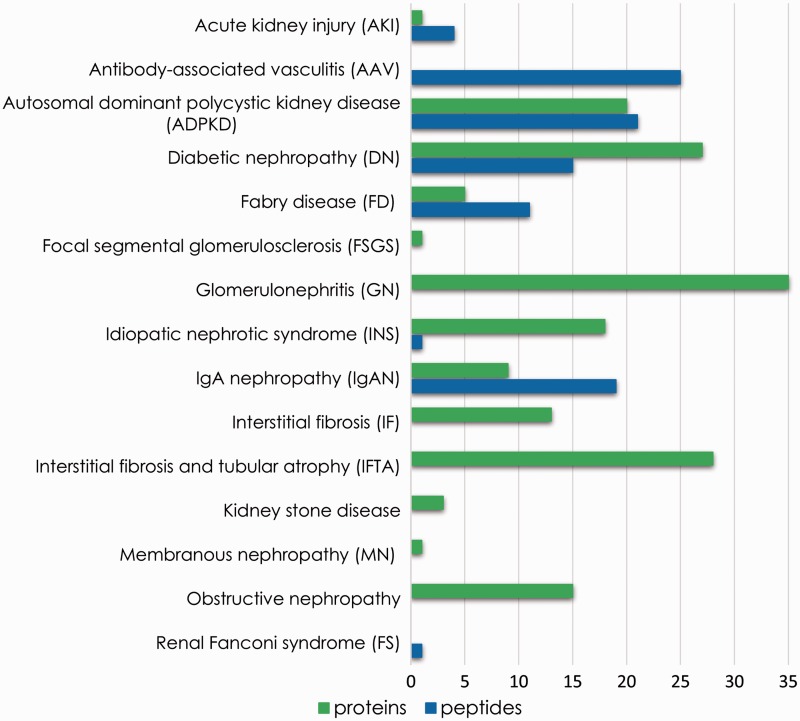
Unique features (peptide sequences and proteins) identified for each disease instance present in the database.

As a proof of concept, we showcase the use of the platform to prove the benefits of meta-analysis of consolidated data or rapid extraction of biomarker information in search for potential biomarkers of chronic kidney disease. Therefore, proteomics and peptidomics profiles were compared, aiming at identification of unique features associated with each disease. These features might be disease-specific putative biomarkers. Figure 5 summarizes the number of unique proteins and peptide sequences associated with each disease reported in the database. Although detailed investigation is needed to assess the feasibility of these substances as markers, this comparison demonstrates that the analysis of integrated data contributes to biomarker research through selection of distinctive features.

**Figure 6.**
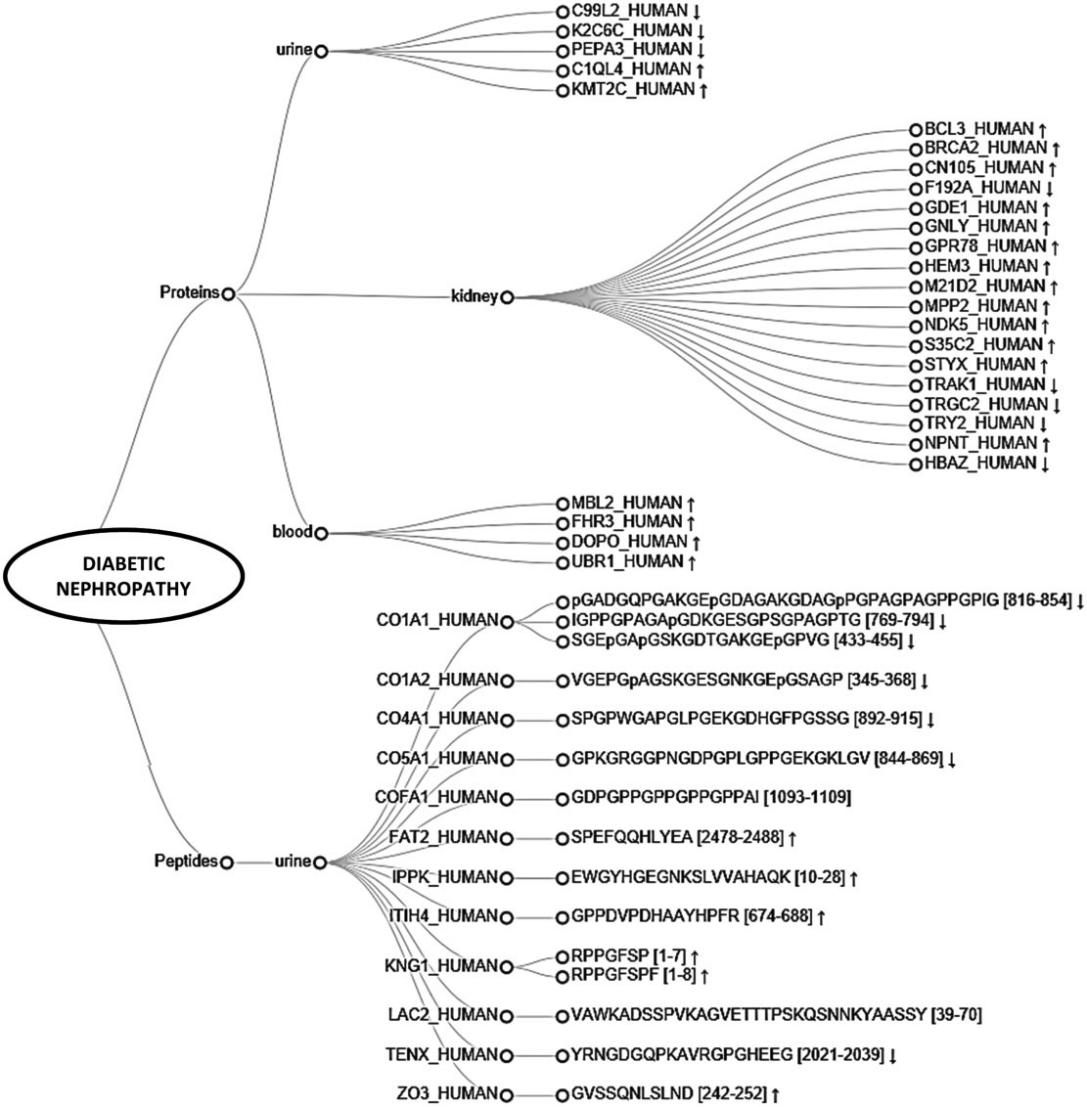
Dendrogram representing peptides and proteins found differentially expressed in different sample types (urine, blood, kidney) and identified as uniquely associated with diabetic nephropathy (DN). Arrows represent reported regulation between cases and controls (↑ -upregulation, ↓ -downregulation).

Furthermore, we explored the unique molecules identified in the studies on diabetic nephropathy (DN). Based on the collected data, 27 proteins and 15 peptide sequences, originating from 12 different proteins were found uniquely associated with DN. Substances are visualized on a dendrogram (Figure 6). Among them kidney (18), urine (5) and plasma (4) proteins, and urine peptides were differentiated (15). Upregulation of majority of kidney derived-proteins suggests the presence of ongoing processes in the kidney. Importantly, among the molecules, six out of seven proteins having catalytic activity [e.g. breast cancer type 2 susceptibility protein (BRCA2), glycerophosphodiester phosphodiesterase 1 (GDE1), MAGUK p55 subfamily member 2 (MPP2)] and 1 transcription factor, i.e. B-cell lymphoma 3 protein (BCL3) are overexpressed. All unique plasma proteins are involved in immune system processes and upregulated, possibly reflecting an overall inflammatory state. Among the urinary proteins, histone-lysine N-methyltransferase 2C (KMT2C) and complement C1q-like protein 4 (C1QL4) might be of interest since they are both overexpressed in DN and involved in regulating fibroblasts proliferation. Considering urinary peptides, extracellular matrix (ECM) components such as collagens are found underrepresented, which is in line with the fact that there is an increased accumulation of ECM in kidney fibrosis (15). Remaining upregulated protein fragments are related to inflammation [i.e. inter-alpha-trypsin inhibitor heavy chain H4 (ITIH4), kininogen-1 (KNG1)] and cellular processes [e.g. protocadherin Fat 2 (FAT2)]. Collectively, molecules identified in the analysis are potentially functionally implicated in characteristic processes related to kidney pathology and might be relevant in relation to diabetic nephropathy. Further investigation is required to assess the validity of those substances as putative biomarkers. Overall, these examples are confirming the usability of the peptiCKDdb database in biomarker screening.

## Discussion and Conclusions

Chronic kidney disease (CKD) is a multi-faceted and highly complex disease, thus elucidation of pathological mechanisms is a real challenge, given the broad range and diversity of distinctive molecular characteristics (16). Based on the high-throughput proteomics experiments, many putative biomarkers of CKD have been identified, shedding some light on the disease processes. However, in majority, their applicability in clinical settings requires further investigation (17). Therefore, data curation and organization is necessary for effective analysis and confirmation of results. Moreover, collective analysis of data across multiple research experiments has a high impact on scientific discovery of biomarkers based on the use of bioinformatics tools (e.g. for statistics, pathway analysis) and systems biology approaches (e.g. multi-omics integration for molecular interactome assembly) (13, 18).

To demonstrate one of the possible applications of the developed platform in biomarker research, we used deposited data to explore the proteomic and peptidomic signatures associated with the underlying causes of CKD. Based on this proof-of-concept, we identified molecular signatures potentially unique, and seemingly functionally relevant, to diabetic nephropathy confirming the usefulness of the established database in rapid extraction of biomarker data and emphasizing the advantages of consolidated data exploration.

The peptiCKDdb database described here is an integrated resource of published peptidomics/proteomics datasets developed to support research efforts in the field of chronic kidney disease. Currently, 114 manually curated datasets are extracted from the published literature on CKD and deposited in the database. Those include studies of body fluids (urine, blood, peritoneal dialysate) and kidney tissue, however, the significantly higher number of the urine-based experiments may originate from the fact that kidney tissue is often hard to obtain, contrary to body fluids. Moreover, current efforts in the biomarker research are focused on the identification of non-invasive markers, thus sample types which are easily accessible are frequently exploited. Comprehensive design of the database structure assures an accurate representation of deposited data. Furthermore, a dedicated web interface enables fast and informative information retrieval. Therefore, we conclude that this development fills the gap in the kidney disease research, being an easy-to-use and efficient tool to manage and mine high-throughput peptidomics and proteomics datasets. Future work will focus mainly on periodic updates, maintenance of the database content and improvement of the interface through the development of data mining tools for initial data analysis.

## Funding

This work was supported by ‘Clinical and system -omics for the identification of the Molecular Determinants of established Chronic Kidney Disease’ (iMODE-CKD; PEOPLE-ITN-GA-2013-608332). Funding for open access charge: iMODE-CKD; PEOPLE-ITN-GA-2013-608332.

*Conflict of interest*. None declared.

## Supplementary data

Supplementary data are available at *Database* Online. 

Supplementary Data
