# Supplementary material for: PeptiCKDdb—peptide- and protein-centric database for the investigation of genesis and progression of chronic kidney disease
Source: Database (Oxford). 2016 Sep 1;2016:baw128. doi: 10.1093/database/baw128 (PMC5009324; doi:10.1093/database/baw128)
Supplement: Supplementary Data [file supp_2016_baw128_index.html]

Supplementary Data 

# PeptiCKDdb—peptide- and protein-centric database for the investigation of genesis and progression of chronic kidney disease

## Supplementary Data

files

- Supplementary Data - zip file
