# Supplementary material for: PeptiCKDdb—peptide- and protein-centric database for the investigation of genesis and progression of chronic kidney disease
Source: Database (Oxford). 2016 Sep 1;2016:baw128. doi: 10.1093/database/baw128 (PMC5009324; doi:10.1093/database/baw128)
Supplement: Supplementary Data [file supp_baw128_suppl_data.zip › Supplementary figures.pptx]

## Slide 1
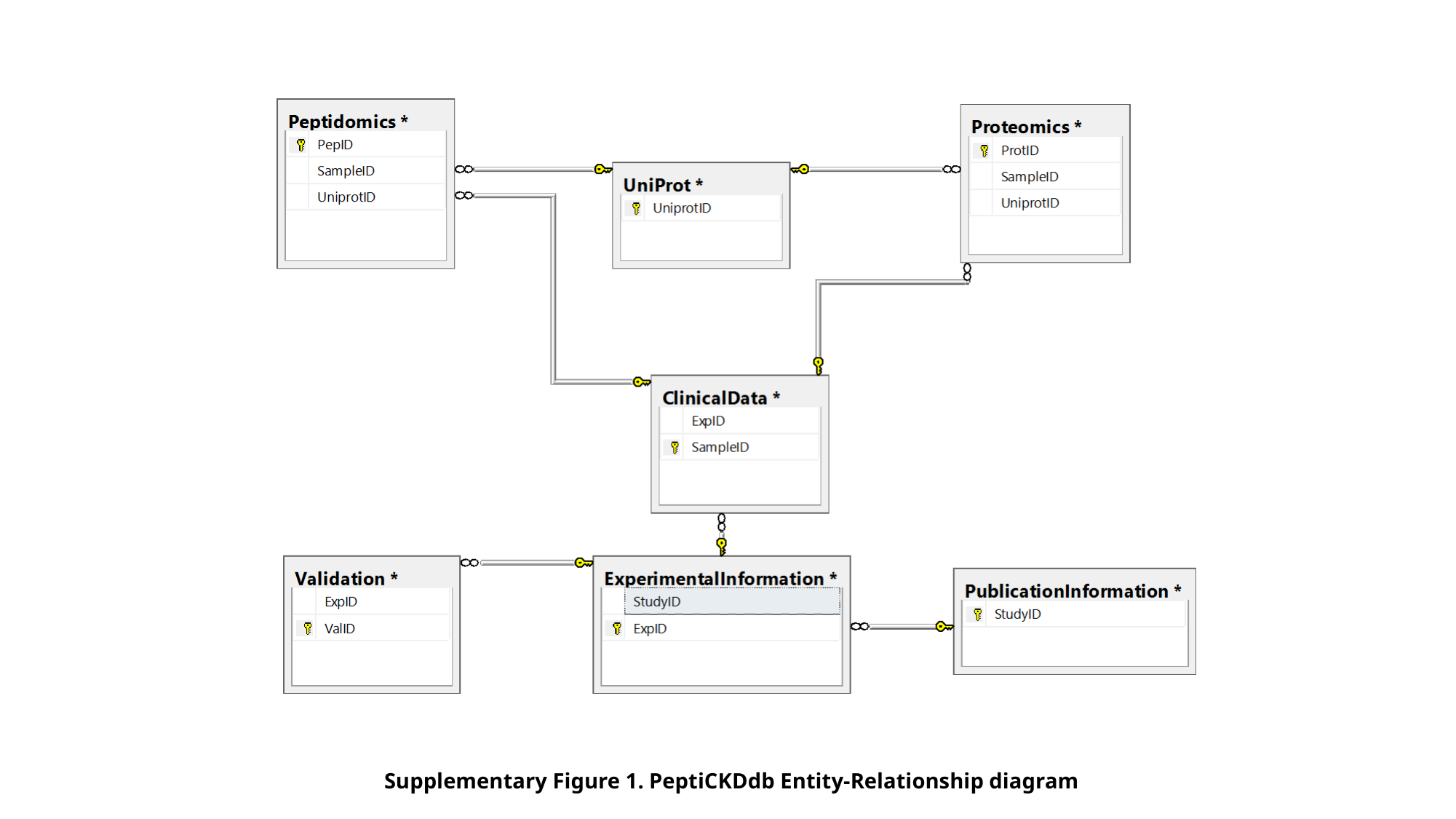

Supplementary Figure 1. PeptiCKDdb Entity-Relationship diagram

## Slide 2
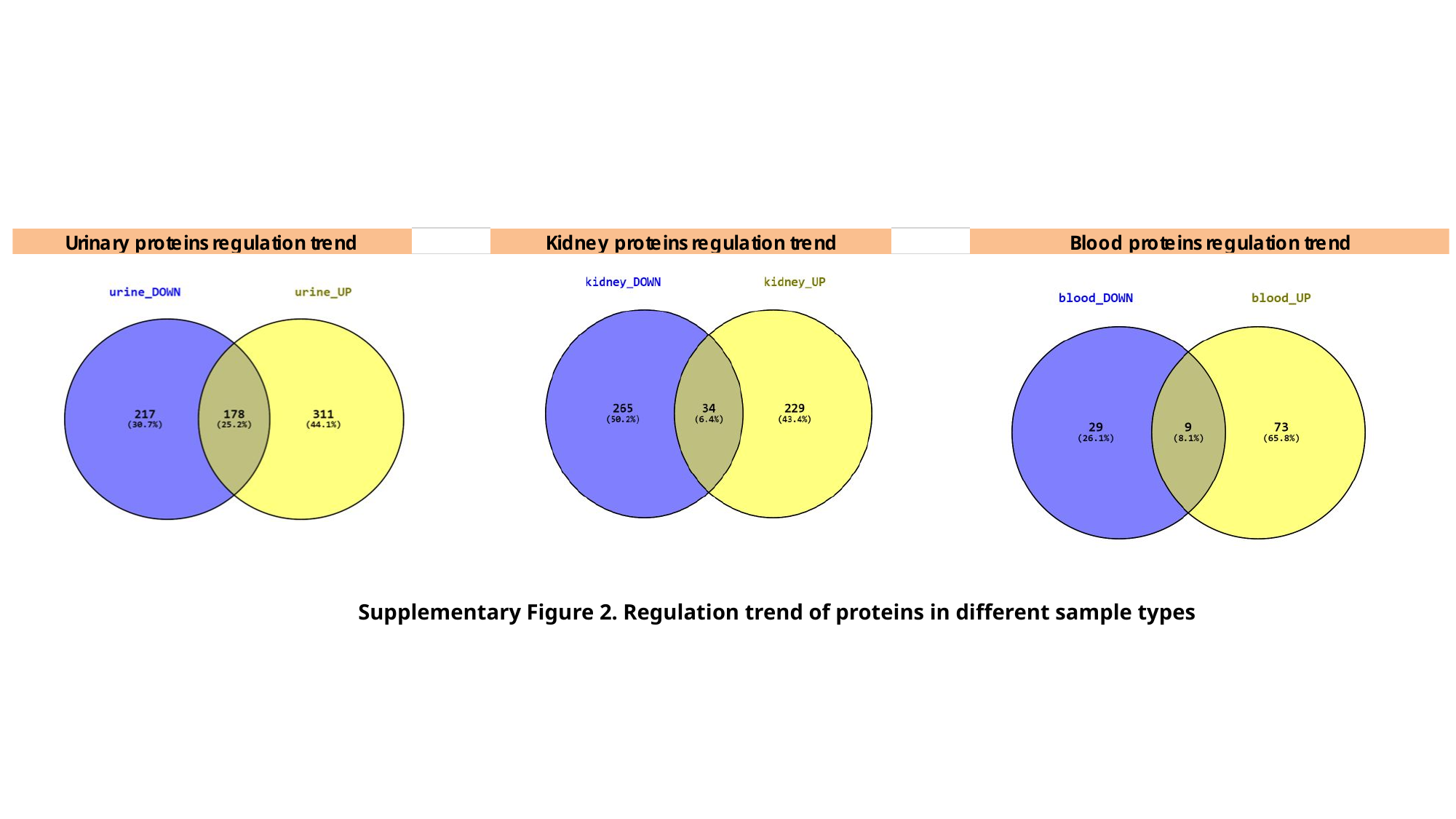

Supplementary Figure 2. Regulation trend of proteins in different sample types

## Slide 3
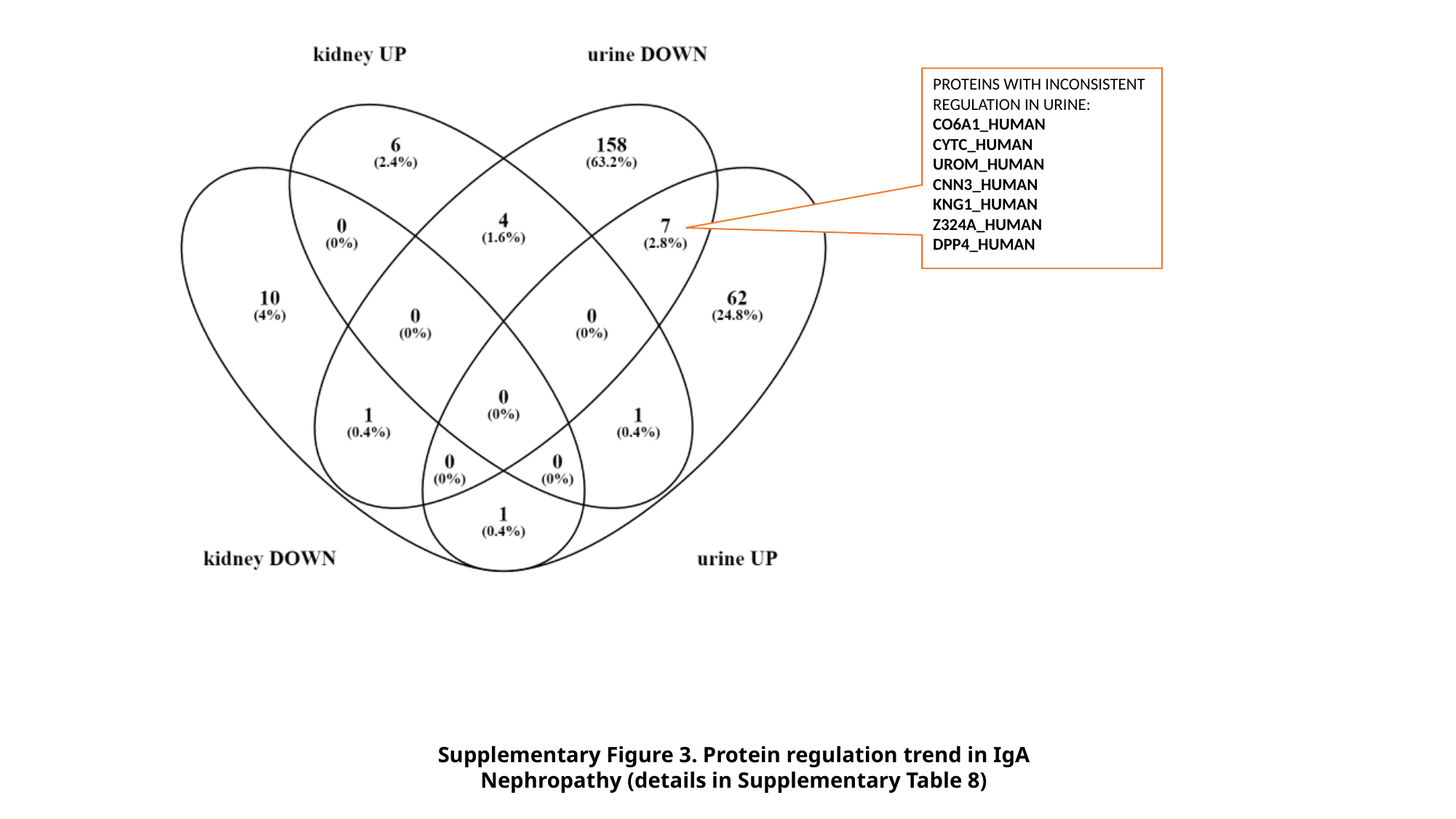

PROTEINS WITH INCONSISTENT REGULATION IN URINE:
CO6A1_HUMAN
CYTC_HUMAN
UROM_HUMAN
CNN3_HUMAN
KNG1_HUMAN
Z324A_HUMAN
DPP4_HUMAN
Supplementary Figure 3. Protein regulation trend in IgA Nephropathy (details in Supplementary Table 8)

## Slide 4
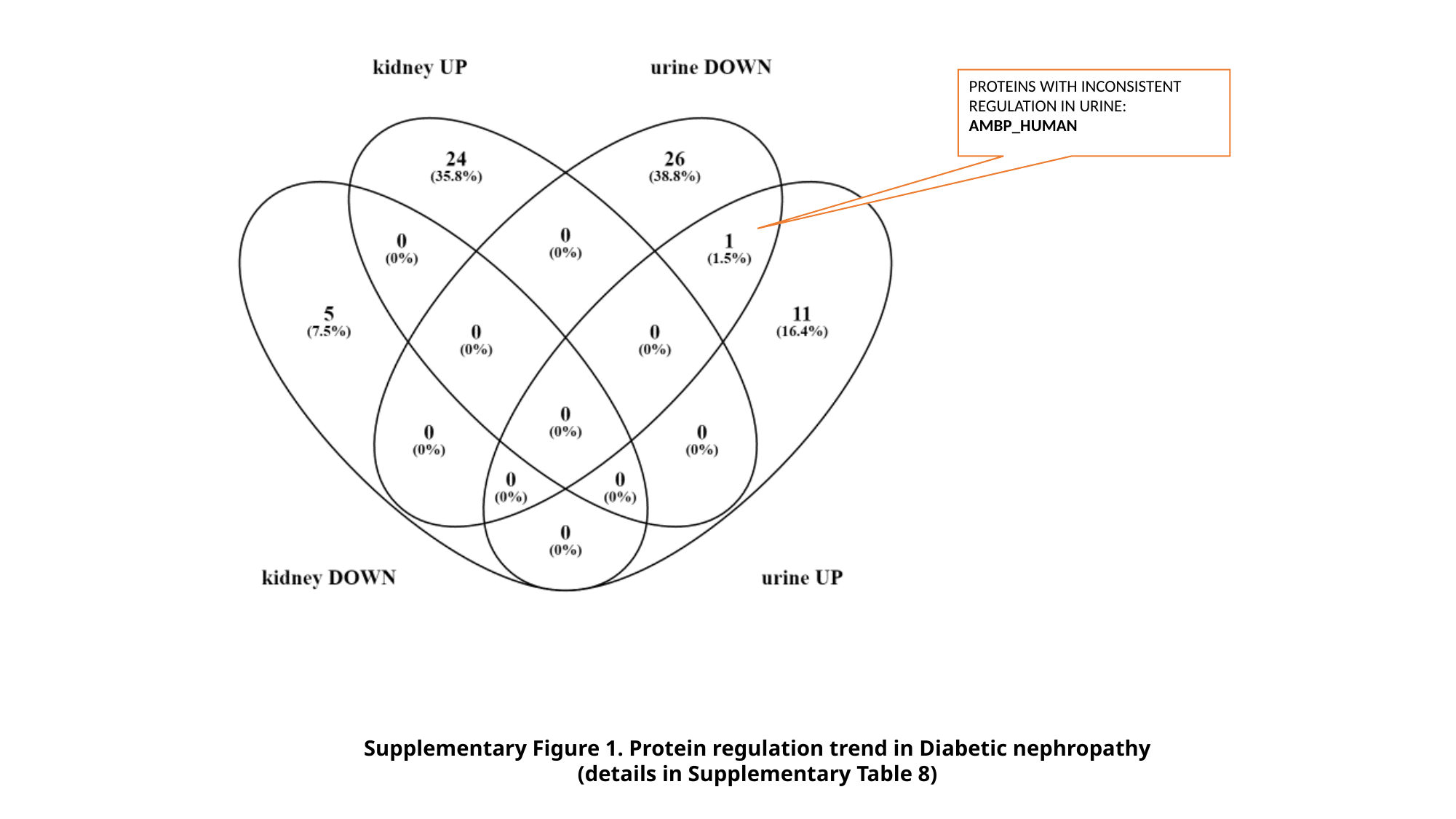

PROTEINS WITH INCONSISTENT REGULATION IN URINE:
AMBP_HUMAN
Supplementary Figure 1. Protein regulation trend in Diabetic nephropathy
(details in Supplementary Table 8)
